# Comparative study of short-term cardiovascular autonomic control in cardiac surgery patients who underwent coronary artery bypass grafting or correction of valvular heart disease

**DOI:** 10.15171/jcvtr.2018.05

**Published:** 2018-03-17

**Authors:** Vladimir A Shvartz, Anton R Kiselev, Anatoly S Karavaev, Kristina A Vulf, Ekaterina I Borovkova, Mikhail D Prokhorov, Andrey D Petrosyan, Olga L Bockeria

**Affiliations:** ^1^Department of Surgical Treatment for Interactive Pathology, Bakulev Scientific Center for Cardiovascular Surgery, Moscow, Russia; ^2^Department of New Cardiological Informational Technologies, Research Institute of Cardiology, Saratov State Medical University, Saratov, Russia; ^3^Department of Nano- and Biomedical Technologies, Saratov State University, Saratov, Russia; ^4^Saratov Branch of the Institute of Radio Engineering and Electronics of Russian Academy of Sciences, Saratov, Russia

**Keywords:** Cardiovascular Autonomic Control, Coronary Artery Bypass Grafting, Valvular Heart Disease, Heart Rate Variability, Slow Oscillations

## Abstract

***Introduction:*** Our aim was to perform a comparative study of short-term cardiovascular autonomic control in cardiac surgery patients who underwent coronary artery bypass grafting (CABG) or surgical correction of valvular heart disease (SCVHD ).

***Methods:*** The synchronous 15 minutes records of heart rate variability (HRV) and finger’s photoplethysmographic waveform variability (PPGV) were performed in 42 cardiac surgery patients (12 women) aged 61.8 ± 8.6 years (mean ± standard deviation), who underwent CABG, and 36 patients (16 women) aged 54.2 ± 14.9 years, who underwent SCVHD , before surgery and in 5-7 days after surgery. Conventional time and frequency domain measures of HRV and index S of synchronization between the slow oscillations in PPGV and HRV were analyzed. We also calculated personal dynamics of these indices after surgery.

***Results: *** We found no differences (*Р * > 0.05) in all studied autonomic indices (preoperative and post-surgery) between studied patients’ groups, except for the preoperative heart rate, which was higher in patients who underwent SCVHD (*P * = 0.013). We have shown a pronounced preoperative and post-surgery variability (magnitude of inter-quartile ranges) of all autonomic indices in studied patients. In the cluster analysis based on cardiovascular autonomic indices (preoperative and post-surgery), we divided all patients into two clusters (38 and 40 subjects) which did not differ in all clinical characteristics (except for the preoperative hematocrit, P = 0.038), index S, and all post-surgery HRV indices. First cluster (38 patients) had higher preoperative values of the HR, TP, HF, and HF%, and lower preoperative values of the LF% and LF/HF.

***Conclusion: ***The variability of cardiovascular autonomic indices in on-pump cardiac surgery patients (two characteristic clusters were identified based on preoperative indices) was not associated with their clinical characteristics and features of surgical procedure (including cardioplegia).

## Introduction

In recent years the attention of researchers has been attracted to heart rate variability (HRV) as a sensitive indicator of the heart autonomic control in cardiac surgery patients.^1^ Lakusic et al^2^ reported that decreased HRV was associated with increased mortality rate in patients who underwent coronary artery bypass grafting (CABG). In contrast, Milicevic et al^3^ has shown that decreased HRV after CABG has no prognostic significance. The value of HRV indices in risk stratification of other cardiac surgery patients is understudied.

Blood pressure variability (BPV) is the second main phenomenon in the cardiovascular autonomic control. BPV is primarily due to the vasomotor tone, which is not directly related to the heart control. Some authors have shown the unchanged vasomotor tone assessed by the low-frequency oscillations in BPV during cardiopulmonary bypass (CPB).^4^ However, the mechanisms of this adaptation require a better understanding. In our previous studies we have used the index of synchronization between the low-frequency oscillations in HRV and photoplethysmographic waveform variability (PPGV) for the assessment of the quality of interaction between the baroreflex control of heart rate and peripheral vascular resistance.^5^ This approach can also be used for studying the cardiovascular adaptation in on-pump cardiac surgery patients.

Cardiac reperfusion injury is the main complication after the following cardioplegic arrest in cardiac surgery patients.^6^ Various approaches have been developed to eliminate the ischemic component of cardioplegia.^7,8^ However, to the best of our knowledge, there are no studies on changes in cardiovascular autonomic control in different on-pump cardiac surgery patients (for example, after CABG or correction of valvular heart disease). A priori hypothesis of our study stated that variants of on-pump cardiac surgery (in the present study, CABG or correction of valvular heart disease), differing from one another in the features of the surgical procedures (cardioplegia, CPB time, etc.; see section “Surgical approach”) and the patients’ baseline clinical status, may have different impact on short-term cardiovascular autonomic control. Studying this question has a potential clinical significance for the personal cardiovascular risk assessment in cardiac surgery patients.

Thus, our aim was to perform a comparative study of short-term cardiovascular autonomic control in cardiac surgery patients who underwent CABG or surgical correction of valvular heart disease (SCVHD).

## Materials and Methods

### Study design

We studied 78 on-pump cardiac surgery patients (28 women) aged 59.8±11.8 years (mean ± standard deviation). 42 cardiac surgery patients (12 women) aged 61.8±8.6 years who underwent CABG, and 36 patients (16 women) aged 54.2±14.9 years who underwent SCVHD.

Patients with abnormalities in heart rate impeding the analysis of HRV, severe heart failure, cardiomyopathy, endocrine pathology except compensated diabetes, cancer, organic diseases in the brain and the nervous system, mental illness, abnormalities in peripheral microcirculation, and symptomatic hypertension were excluded from the study.

All the patients underwent clinical examination, electrocardiography, echocardiography, and blood test.

### Surgical approach

The standard operative technique was applied to all the patients using CPB (on-pump).

On-pump CABG was conducted on a beating heart, that is, in the parallel perfusion and normothermia, without aortic cross clamping and the use of cardioplegic solution. CPB was performed through the aortic cannulation and installing a single two-stage cannula into the right atrium through the free wall. As the conduits, the left internal thoracic artery (or sometimes the internal thoracic artery from the right side), radial artery, and great saphenous vena were used. To stabilize the myocardium at the coronary anastomoses formation stage, the vacuum stabilizers of the myocardium “Octopus” (Medtronic, USA) or “Acrobat” (Maquet, Germany) were used. All the patients after the completion of CABG, in the absence of contraindications, underwent intraoperative graft angiography in order to assess the quality of formed anastomoses. The surgery was finished in a standard way using drainage of the pericardial cavity and the anterior mediastinum and stratified suturing wounds of the chest.

On-pump SCVHD was conducted in hypothermia and pharmacological cardioplegia. It was carried out through the aortic cannulation, separate cannulation of the superior vena cava using angled cannula, and direct cannulation of the inferior vena cava via the free wall of the right atrium. Cardioplegia was performed antegrade in the aortic root or, in case of the insufficiency of the aortic valve, retrograde through the coronary sinus. For cardioplegia, the cardioplegic solutions Custodiol (Odyssey Pharmaceuticals Inc., Germany) were employed. The surgery was finished in a standard way using drainage of the pericardial cavity and the anterior mediastinum.

The duration of CPB was 73 (56, 98) minutes in patients who underwent CABG and 124 (102, 145) minutes in patients who underwent SCVHD (*P*<0.001). The data are presented as median with inter-quartile range (Table 1). In patients who underwent SCVHD the duration of aortic cross clamping was 65 (57, 75) minutes. None of the patients had signs of perioperative myocardial infarction.

### Autonomic control acquisition and processing

To examine the cardiovascular autonomic control, we carried out the analysis of HRV and estimated the degree of synchronization between the slow oscillations in HRV and PPGV.

The synchronous 15-minutes records of R-R interval (RRI) time series of heart rate and photoplethysmogram (PPG) measured on the middle finger of the hand were performed in all patients before surgery and 5-7 days after surgery. The patients were investigated in the morning under spontaneous breathing. All signals were sampled at 250 Hz and digitized at 14 bits. We excluded from the analysis the series with forced inspiration and delays in breathing. For further analysis, only RRIs and PPG records without artifacts, extrasystoles, and considerable trends were exploited.

**Figure 1:**
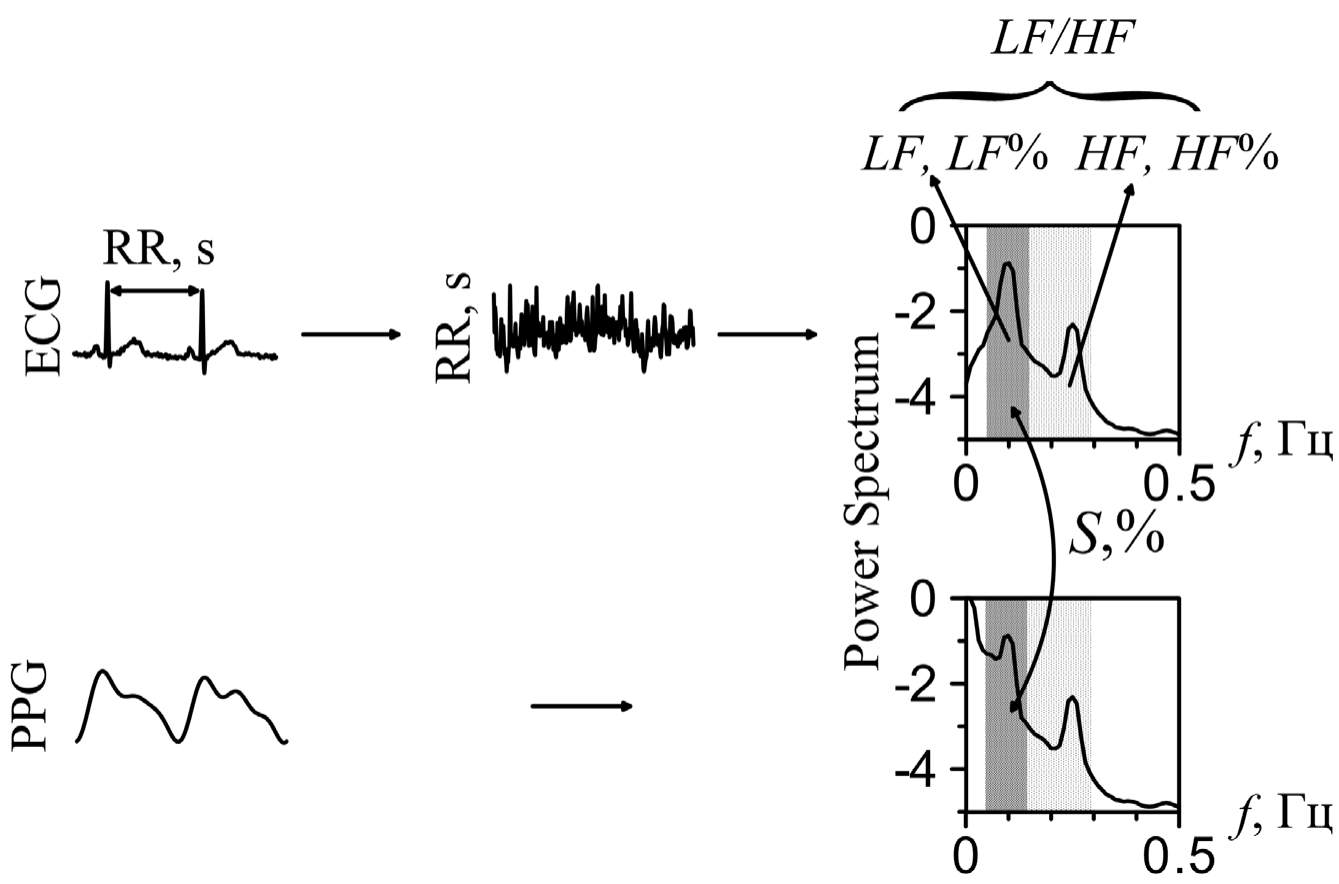
Illustration of the technique used in the article, which includes the following steps: registration of experimental ECG and PPG signals, extraction of the sequence of RR intervals from the ECG, evaluation of the individual activity of circuits of autonomous regulation of blood circulation by calculating the HRV spectral density in LF and HF ranges, calculating the normalized indices (LF%, HF%), an estimation of the degree of interaction of the elements of autonomous regulation with the analysis of the index S between the spectral components of HRV and PPG in the LF-band.

To estimate the synchronization between the slow oscillations in HRV and PPGV we applied the method proposed by us recently.^5^ Index *S* defines the relative time (in percents) of synchronization between the considered low-frequency oscillations (Figure 1). We also calculated the following assessments of HRV: heart rate (HR, bmp), standard deviations of normal to normal intervals (SDNN, ms), total power of HRV spectrum (0 to 0.5 Hz) (TP, ms^2^), power of low-frequency (LF, ms^2^; 0.04 to 0.15 Hz) and high-frequency (HF, ms^2^; 0.15 to 0.40 Hz) components of HRV spectrum, the ratio of LF to TP (LF%), the ratio of HF to TP (HF%), the ratio of LF to HF (LF/HF).^9^ In each patient, we calculated the personal dynamics of these indices after the surgery denoted as ΔS, ΔHR, ΔSDNN, ΔTP, ΔHF%, ΔLF%, and ΔLF/HF. For example, ΔS = S_after surgery_ ‒ S_preoperative_).

### Statistical analysis

Continuous variables are reported as medians (Me) with low and upper quartiles (LQ, UQ). Binary data are presented as frequencies and percentages. The obtained estimations were considered statistically significant, if *P* was less than 0.05. For the statistical analysis, the software package Statistica 6.1 (StatSoft Inc., Tulsa, Oklahoma, USA) was used.

We applied the Shapiro–Wilk test to check whether the HRV spectral data are approximately normally distributed. Since these data are non-normal, their further analysis was carried out using nonparametric statistical methods. To compare the continuous variables, we used the Mann–Whitney test. To compare the variables within one patients’ group we used the Wilcoxon test. To compare the two proportions, we used two-sided z-test.

To separate the studied patients into groups in accordance with the values of their autonomic indices, we used the cluster analysis (k-means clustering). The following factors (measured before and after the surgery) were considered in the cluster analysis: index S, HR, SDNN, TP, LF, HF, LF%, HF%, and LF/HF. In this analysis personal dynamics of the following indices after the surgery were also included: ΔS, ΔHR, ΔSDNN, ΔTP, ΔHF%, ΔLF%, and ΔLF/HF.

## Results

### Baseline clinical characteristics of patients

Baseline clinical characteristics of patients are shown in Table 1. Patients who underwent CABG had higher values of the following baseline clinical parameters: body mass index, diastolic blood pressure, creatinine, frequencies of prior myocardial infarction, diabetes and hypertension, frequencies of prior percutaneous coronary interventions, frequencies of preoperative treatment with angiotensin-converting enzyme (ACE) inhibitors, beta-blockers, calcium antagonists, and statins. Patients who underwent SCVHD had higher values of left ventricular ejection fraction, CPB time, and frequency of any in-hospital complications (acute coronary syndrome, acute renal failure, cardiac arrest, cardiac reoperation for bleeding, cardiac reoperation other than bleeding, deep sternal wound infection, stroke, and pneumonia). In studied groups, there was no in-hospital mortality.

**Table 1:** Baseline clinical characteristics of patients in the studied groups

**Parameters**	**Data format**	**Patients ongoing CABG (n=42)**	**Patients ongoing SCVHD (n=36)**	***P*** ** level**
Age, y	Me (LQ, UQ)	63 (57, 67)	58 (47, 65)	0.086
Male sex	No. (%)	30 (71.4%)	20 (55.6%)	0.152
BMI, kg/m^2^	Me (LQ, UQ)	29.2 (27.3, 32.9)	25.6 (23.2, 29.0)	0.001
Prior MI	No. (%)	30 (71.4%)	0 (0)	<0.001
Prior stroke	No. (%)	2 (4.8%)	2 (5.6%)	0.874
Hypertension	No. (%)	40 (95.2%)	18 (50.0%)	<0.001
Smoking	No. (%)	15 (35.7%)	6 (16.7%)	0.063
Diabetes	No. (%)	7 (16.7%)	0 (0)	0.012
COPD	No. (%)	3 (7.1%)	0 (0)	0.107
Prior PCI	No. (%)	8 (19.0%)	0 (0)	0.007
Baseline SBP, mm Hg	Me (LQ, UQ)	130 (120, 130)	120 (110, 130)	0.029
Baseline DBP, mm Hg	Me (LQ, UQ)	80 (70, 85)	78 (70, 80)	0.014
Baseline LVEF, %	Me (LQ, UQ)	60 (55, 62)	66 (60, 67)	0.001
LVEF in 5-7 days after surgery, %	Me (LQ, UQ)	59 (54, 64)	64 (60, 66)	0.002
**Preoperative Blood Test**				
Creatinine, mg/dL	Me (LQ, UQ)	84 (74, 105)	69 (61, 88)	0.044
Glucose, mmol/L	Me (LQ, UQ)	5.4 (4.9, 6.2)	5.0 (4.7, 5.2)	0.051
Leukocytes, *10^9^/mL	Me (LQ, UQ)	7.4 (6.0, 8.8)	6.9 (5.4, 7.2)	0.315
Lactate, mmol/L	Me (LQ, UQ)	0.9 (0.8, 1.3)	0.8 (0.7, 1.3)	0.813
Hematocrit, %	Me (LQ, UQ)	41 (38, 44)	40 (37, 42)	0.779
**Previous Treatment**				
ACE inhibitors	No. (%)	34 (81.0%)	10 (27.8%)	<0.001
Beta-blockers	No. (%)	34 (81.0%)	8 (22.2%)	<0.001
Statins	No. (%)	30 (71.4%)	8 (22.2%)	<0.001
Diuretics	No. (%)	15 (35.7%)	16 (44.4%)	0.436
Calcium antagonists	No. (%)	10 (23.8%)	2 (5.6%)	0.029
**Treatment After Surgery**				
ACE inhibitors	No. (%)	36 (85.7%)	15 (41.7%)	<0.001
Beta-blockers	No. (%)	38 (90.5%)	10 (27.8%)	<0.001
Statins	No. (%)	40 (95.2%)*	8 (22.2%)	<0.001
Diuretics	No. (%)	16 (38.1%)	12 (33.3%)	0.661
Calcium antagonists	No. (%)	12 (28.6%)	2 (5.6%)	0.010
**Features of Surgery and Postoperative Period**				
Cardioplegia	No. (%)	0 (0 %)	36 (100%)	<0.001
CPB time, minutes	Me (LQ, UQ)	73 (56, 98)	124 (102, 145)	<0.001
ACCT, minutes	Me (LQ, UQ)	―	65 (57, 75)	―
PMVT, hours	Me (LQ, UQ)	13.5 (9.0, 21.5)	20.0 (13.0, 25.0)	0.099
Length of ICU stay, days	Me (LQ, UQ)	1.0 (1.0, 1.0)	1.0 (1.0, 1.0)	0.606
Length of hospital stay, days	Me (LQ, UQ)	7.5 (7.0, 8.0)	8.0 (7.0, 11.0)	0.085
Any in-hospital complication	No. (%)	7 (16.7%)	14 (38.9%)	0.039

Continuous variables are presented as medians with inter-quartile ranges – Me (LQ, UQ). Categorical data are presented as frequencies and percentages – No. (%). * Significant differences (*P* < 0.05) from level of similar parameter before surgery. CABG, coronary artery bypass grafting; SCVHD, surgical correction of valvular heart disease; BMI, body mass index; MI, myocardial infarction; COPD, chronic obstructive pulmonary disease; PCI, percutaneous coronary intervention; SBP, systolic blood pressure; DBP, diastolic blood pressure; LVEF, left ventricular ejection fraction; ACE, angiotensin-converting enzyme; CPB, cardiopulmonary bypass; ACCT, aortic cross clamping time; PMVT, postoperative mechanical ventilation time; ICU, intensive care unit.

### Cardiovascular autonomic indices before and after on-pump cardiac surgery

We found no differences (*Р *> 0.05) in all parameters (preoperative and post-surgery) of cardiovascular autonomic control between both groups of patients (who underwent CABG or SCVHD) except for the preoperative HR (Table 2). Preoperative HR was higher in patients who underwent SCVHD (*P*=0.013). We revealed a pronounced variability of most autonomic indices and post-surgical dynamics of these indices in both patient groups (see magnitude of inter-quartile ranges in Table 2).

**Table 2:** Cardiovascular autonomic indices before and in 5-7 days after the cardiac surgery

**Parameters**	**Patients ongoing CABG (n=42)**	**Patients ongoing SCVHD (n=36)**	***P*** ** value**
**Preoperative Values**			
Index S, %	24.7 (18.3, 35.2)	22.5 (13.4, 27.4)	0.400
HR, bmp	67 (60, 72)	69 (65, 80)	0.013
SDNN, ms	34.9 (24.9, 52.3)	37.7 (28.4, 53.1)	0.903
TP, ms^2^	493 (311, 1247)	425 (246, 969)	0.696
LF, ms^2^	134 (47, 315)	135 (83, 248)	0.570
HF, ms^2^	123 (25, 508)	74 (33, 188)	0.428
LF%	24.4 (16.5, 34.6)	29.4 (17.1, 36.9)	0.653
HF%	21.6 (9.5, 47.0)	20.2 (11.1, 29.7)	0.422
LF/HF	1.2 (0.6, 2.7)	1.4 (0.6, 2.9)	0.546
**5-7 Days After the Cardiac Surgery**			
Index S, %	20.3 (10.2, 27.5)	20.2 (15.5, 28.1)	0.529
HR, bmp	78 (73, 87)*	74 (66, 90)	0.439
SDNN, ms	15.6 (11.3, 41.0)	18.0 (11.1, 71.9)	0.479
TP, ms^2^	84 (26, 496)*	125 (36, 2735)	0.326
LF, ms^2^	27 (7, 150)	53 (9, 637)	0.446
HF, ms^2^	11 (5, 201)	24 (4, 893)	0.663
LF%	26.6 (18.1, 36.4)	30.8 (21.8, 39.8)	0.526
HF%	26.3 (10.8, 46.4)	23.7 (9.7, 43.4)	0.637
LF/HF	0.8 (0.5, 3.4)	1.2 (0.6, 2.9)	0.586
**Personal Dynamics of Indices**			
ΔS, %	-3.1 (-19.0, +5.8)	+0.5 (-14.2, +6.3)	0.488
ΔHR, bpm	+13 (+1, +28)	+3 (-6, +15)	0.144
ΔSDNN, ms	-16.0 (-38.9, +17.6)	-14.9 (-26.1, +6.3)	0.637
ΔTP, ms^2^	-293 (-841, -44)	-194 (-640, +234)	0.304
ΔLF%	+3.4 (-12.6, +12.3)	+1.6 (-12.4, +14.9)	0.977
ΔHF%	-0.5 (-13.8, +9.2)	+1.7 (-16.8, +24.0)	0.783
ΔLF/HF	+0.2 (-1.6, +1.1)	0 (-1.1, +2.5)	0.532

Data are presented as medians with inter-quartile ranges – Me (LQ, UQ). Index S is relative time (in percents) of synchronization between low-frequency oscillations in HRV and PPGV. * Significant differences (*P* < 0.05) from level of similar parameter before surgery. CABG, coronary artery bypass grafting; SCVHD, surgical correction of valvular heart disease; HR, heart rate; SDNN, standard deviations of normal to normal intervals; TP, total power (0 to 0.5 Hz) of HRV spectrum; LF, power of low-frequency band (0.04 to 0.15 Hz) of HRV spectrum; HF, power of high-frequency (0.15 to 0.40 Hz) band of HRV spectrum; LF%, ratio (in percents) of LF to TP; HF%, ratio (in percents) of HF to TP; LF/HF, ratio of LF to HF; ΔS = S_after surgery_ ‒ S_preoperative_; ΔHR = HR_after surgery_ ‒ HR_preoperative_; ΔSDNN = SDNN_after surgery_ ‒ SDNN_preoperative_; ΔTP = TP_after surgery_ ‒ TP_preoperative_; ΔLF% = LF%_after surgery_ ‒ LF%_preoperative_; ΔHF% = HF%_after surgery_ ‒ HF%_preoperative_; ΔLF/HF = LF/HF_after surgery_ ‒ LF/HF_preoperative_.

After cardiac surgery, some indices of HRV (SDNN, TP, LF, and HF) were decreased in most studied patients (see median and quartiles of these indices and their personal dynamics in Table 2). In a part of on-pump cardiac surgery patients, we revealed the increase of most HRV indices (see upper quartile of distributions of their personal dynamics in Table 2).

### Results of cluster analysis

In the cluster analysis, we divided all studied patients (n=78) into two groups according to the similarity of their cardiovascular autonomic indices (preoperative and post-surgery). The first group (named as Cluster 1) contains 38 patients, while the second group (named as Cluster 2) includes 40 patients. Clinical characteristics and autonomic indices of patients in Clusters 1 and 2 are shown in Table 3, in which only statistically significant differences between the Clusters are presented.

**Table 3:** Clinical characteristics and autonomic indices of patients in the selected clusters

**Parameters**	**Cluster 1 (n=38)**	**Cluster 2 (n=40)**	***P*** ** value**
Preoperative hematocrit, %	42 (38, 44)	39 (36, 41)	0.038
**Preoperative autonomic indices**			
HR, bpm	70 (62, 78)	65 (58, 71)	0.046
TP, ms^2^	768 (311, 2385)	356 (270, 738)	0.048
HF, ms^2^	274 (125, 1127)	34 (21, 73)	<0.001
LF%	20.4 (14.7, 35.7)	32.3 (21.1, 36.6)	0.047
HF%	40.1 (27.3, 59.4)	10.1 (5.4, 15.6)	<0.001
LF/HF	0.6 (0.3, 1.0)	2.9 (1.9, 4.5)	<0.001
**Personal dynamics of indices**			
ΔHR, bpm	-1 (-10, +15)	+13 (+4, +24)	0.018
ΔLF%	+7.6 (0, +12.5)	-1.1 (-13.5, +6.9)	0.049
ΔHF%	-14.0 (-24.5, -3.1)	+16.5 (+2.0, +34.1)	<0.001
ΔLF/HF	+0.5 (+0.2, +2.6)	-1.7 (-3.2, -0.4)	<0.001

Data are presented as medians with inter-quartile ranges – Me (LQ, UQ). We presented only parameters (clinical, autonomic) with statistically significant (*P*<0.05) differences between the Clusters. HR, heart rate; SDNN, standard deviations of normal to normal intervals; TP, total power (0 to 0.5 Hz) of HRV spectrum; HF, power of high-frequency (0.15 to 0.40 Hz) band of HRV spectrum; LF%, ratio (in percents) of LF to TP; HF%, ratio (in percents) of HF to TP; LF/HF, ratio of LF to HF; ΔHR = HR_after surgery_ ‒ HR_preoperative_; ΔLF% = LF%_after surgery_ ‒ LF%_preoperative_; ΔHF% = HF%_after surgery_ ‒ HF%_preoperative_; ΔLF/HF = LF/HF_after surgery_ ‒ LF/HF_preoperative_.

We found no differences (*Р*>0.05) in all clinical characteristics (including ratio of patients who underwent CABG or SCVHD, aortic cross clamping time, CBP time, frequency of any in-hospital complications, length of intensive care unit stay and hospital stay, etc.) between Cluster 1 and Cluster 2, except for the preoperative hematocrit (*P*=0.038). In selected Clusters patients also had no differences (*Р*>0.05) in index *S* (preoperative and post-surgery) and all post-surgery HRV indices. The patients in Cluster 1 had higher values of the following preoperative parameters (Table 3): hematocrit, HR, TP, HF, and HF%. The patients in Cluster 2 had higher values of preoperative LF% and LF/HF ratio (Table 3).

## Discussion

The problem of using HRV in risk stratification of cardiac patients is still actual for the modern cardiology.^10^ The predictive value of some HRV parameters such as SDNN and non-linear indices is well known for the risk assessment in patients after myocardial infarction and patients with left ventricular dysfunction.^11,12^ However, the impact of HRV indices on the risk stratification and autonomic dysfunction assessment in cardiac surgery patients is not well known yet.

CABG is associated with a noticeably decreased HRV in the early postoperative period. Thus, the long-term predictive value of HRV indices may be lost in patients after CABG.^3,13^ Nevertheless, some authors reported well post-surgical predictive value of non-linear HRV parameters in patients after CABG.^14,15^ Changes in the autonomic control of the heart, associated with CABG, lead to unpredictable cardiovascular events during and after surgery.^16,17^ In particular, the increased HF% and peak point correlation dimension before CABG are associated with postoperative atrial fibrillation.^18^ These statements are not confirmed by our findings on short-term follow-up in the present study.

In patients with valvular heart disease, the decreased HRV is caused by a dysfunction of ventricular baroreceptors against the background of pressure overload of the right heart.^19,20^ Surgical correction of valve defect may remove this factor.

We observed a post-surgery decrease of HRV in most on-pump cardiac surgery patients (see SDNN, TP, and their personal dynamics in Table 2) which is consistent with other studies.^19-22^ These changes in autonomic control are probably due to peculiarities of surgical procedure (anesthesia, CPB time, aortic cross clamping time, cardioplegia, etc.).^21,23,24^ However, current knowledge about the association between the peculiarities of surgical procedure and post-surgery events caused by the autonomic dysfunction is contradictory.^25-27^ Note that in a part of on-pump cardiac surgery patients included in our study, we revealed the increase of HRV (see upper quartile of distributions of ΔSDNN, and ΔTP in Table 2) which differs from the results of other authors. We found no association between autonomic control indices and clinical characteristics of patients including the features of surgical procedure (see Tables 2 and 3).

Almost all studied preoperative variables (age, body mass index, old myocardial infarction, hypertension, left ventricular ejection fraction, beta-blockers, etc.) can influence HRV.^9^ However, we did not find associations of differences between patients on preoperative parameters and autonomic indices.

Souza Neto et al^4^ showed that slow BPV did not change during CPB in patients receiving no preoperative treatment for cardiovascular diseases, in contrast to patients receiving preoperative treatment with angiotensin-converting enzymes and beta-blockers. In our study the patients divided into two groups according to the features of their autonomic status have no differences in drug treatment (see the Section “Results of cluster analysis”).

Conventional HRV indices approved by the European Society of Cardiology and the North American Society of Pacing and Electrophysiology ^9^ do not allow to properly evaluate the dynamics and non-linear properties of cardiovascular autonomic control. Therefore, many authors exploit a non-linear approach to the HRV assessment.^28,29^ However, HRV allows evaluating only the autonomic control of the heart rhythm. Additional information on cardiovascular autonomic control can be obtained from PPGV characterizing the fluctuations in peripheral blood flow. Slow oscillations in PPGV have potentially important clinical applications for risk stratification and autonomic dysfunction assessment in patients.^30^ In our previous studies we have shown the clinical efficiency of the analysis of synchronization of low-frequency oscillations in HRV and finger’s PPGV for the risk stratification in patients with acute myocardial infarction, and autonomic dysfunction assessment during treatment in hypertensive patients and patients with coronary artery disease.^5,31,32,33^ In the present study the preoperative and post-surgery distributions of the index of synchronization of slow oscillations in HRV and PPGV were similar in both studied patients’ groups (Table 2).

We revealed the similar variability of preoperative status of cardiovascular autonomic control and its post-surgical dynamics in patients who underwent CABG and patients who underwent SCVHD (see Table 2). The statistically significant difference in preoperative HR between CABG and SCVHD patients (see Table 2) does not seem to be clinically significant: 69 (65, 80) in SCVHD vs 67 (60,72) in CABG. The Clusters selected according to the similarity of autonomic control indices were not associated with the type of cardiovascular surgical pathology (see the Section “Results of cluster analysis”). The selected Clusters of on-pump cardiac surgery patients have significant differences in preoperative autonomic control indices, and have no differences in post-surgical level of these indices. In simple terms, 5 to 7 days after on-pump cardiac surgery some patients restored their preoperative cardiovascular autonomic control more completely and some less so. The clinical significance of this fact requires an examination in future studies.

## Conclusion

We found no differences in preoperative and post-surgery cardiovascular autonomic indices (except for the preoperative HR) between patients who underwent CABG and patients who underwent SCVHD. The revealed variability of cardiovascular autonomic indices in the studied cardiac surgery patients was not associated with their clinical characteristics, features of surgical procedure, and early postoperative outcomes.

## Study limitations

Our study included only 36 patients who underwent SCVHD and 42 patients who underwent CABG. These groups are rather small. Therefore, we did not use multiple regression to study the short-term cardiovascular autonomic control in cardiac surgery patients. We used only cluster analysis, which somewhat limits our results.

A lack of long-term follow-up and small number of patients are the main limitations for establishing the predictive value of the obtained results on the dynamics of cardiovascular autonomic indices in patients who undergo CABG or SCVHD.

Two studied patient groups (who underwent CABG or SCVHD) differ substantially. Since valvular heart disease and valve surgery have always been associated with cardioplegia use, it remains unclear which of these two factors led to any observed consequences. Note that all patients without cardioplegia were treated for coronary artery disease. But we found no differences in all preoperative and post-surgery cardiovascular autonomic indices between these groups. Since all studied patients underwent on-pump cardiac surgery, we conditionally regarded them as one summary group.

Two additional limitations are characteristic of our study: (*i*) we did not study diurnal variation of cardiovascular autonomic indices; (*ii*) we used short time records of RRIs and PPG.

## Competing interests

The authors confirm that this article content has no conflict of interest.

## Ethical approval

This observational prospective study was approved by the Ethics Committee of the Bakulev Scientific Center for Cardiovascular Surgery in Moscow, Russia, and informed consent was obtained from all participants. All procedures performed in studies involving human participants were in accordance with the ethical standards of the institutional research committee and with the 1964 Helsinki Declaration and its later amendments or comparable ethical standards.
